# Study on the Knowledge, Attitude, and Practice (KAP) of Nursing Staff and Influencing Factors on COVID-19

**DOI:** 10.3389/fpubh.2020.560606

**Published:** 2021-01-18

**Authors:** Xin Wen, Fan Wang, Xiuyang Li, Hua Gu

**Affiliations:** ^1^Zhejiang Provincial Center for Medical Science and Education Development, Hangzhou, China; ^2^Clinical Big Data and Statistics Center, Second Affiliated Hospital, Zhejiang University College of Medicine, Hangzhou, China; ^3^Department of Epidemiology and Biostatistics, Zhejiang University, Hangzhou, China

**Keywords:** COVID-19, knowledge, attitude, practice, nursing staff, influencing factors

## Abstract

The aim of this study is to investigate the knowledge, attitude, and practice (KAP) on Coronavirus Disease 2019 (COVID-19) care among nursing staff and analyze its influencing factors. The survey was conducted on February 18, 2020, among 7,716 voluntary participants from 143 medical institutions in Zhejiang, China. The findings indicated that KAP of nursing staff scored well. However, the accuracy of psychological nursing knowledge was much lower, 14.3% only. Nursing staff working in isolation wards have higher knowledge (OR = 1.776, 95% CI: 1.491–2.116), attitude (OR = 1.542, 95% CI: 1.298–1.832), and practice (OR = 1.902, 95% CI: 1.590–2.274) scores than those in general wards. In terms of KAP, nursing staff with working experience ≤ 10 years scored lower than those with working experience ≥ 20 years, with OR values of 0.490 (95% CI: 0.412–0.583), 0.654 (95% CI: 0.551–0.775), and 0.747 (95% CI: 0.629–0.886), respectively. It is necessary to take measures to enhance the training on COVID-19, especially for KAP of junior nurses in general wards.

## Introduction

At the end of December 2019, a novel coronavirus pneumonia case appeared in Wuhan, China (1). The World Health Organization (WHO) named it Coronavirus Disease 2019 (COVID-19) and declared it a pandemic (2). As of April 29, 2020, there were nearly 3 million confirmed cases of COVID-19, including 202,733 deaths according to the WHO, reported by 213 countries around the world (3).

Zhejiang province locates on the east coast of China, which was one of the regions hit hardest when COVID-19 broke out in China. It is located between 118°01′-123°10′ E and 27°02′-31°11′ N, is economically developed, with an area of 105,500 km^2^ and a population of about 50 million, and consists of 11 municipalities. The first case of COVID-19 reported by the Health Commission of Zhejiang Province was on January 21, 2020. As of February 18, 2020, when we conducted the survey, a total of 1,173 confirmed cases were reported in Zhejiang province and 544 were discharged from hospitals (4).

The model of KAP is divided into three continuous processes that help to explain human behavior. As a new respiratory infectious disease, COVID-19-related nursing care involves new knowledge and contents. Attitude and practice of nursing staff also affect the progression and prognosis of the disease. The purpose of the study is to understand the nursing staffs' knowledge, attitude, and practice (KAP) on COVID-19 and its influencing factors, which can provide a basis for further targeted improvement measures, and provide references for subsequent relevant training or policy-making to improve the quality of clinical nursing.

## Materials and Methods

### Participants

We organized an anonymous and online survey on COVID-19 nursing for all nursing staff in our province through the questionnaire star, a mobile app, which greatly improved the efficiency of survey. A total of 7,716 nursing staff from 143 medical institutions volunteered to participate in the survey, which includes 11 medical institutions at the provincial level, 53 institutions at the municipal level, and 79 institutions at the county level. The survey was conducted on February 18, 2020, 68 days after the report of the first case in China (December 12, 2019).

### Survey Instrument

The self-designed questionnaire includes basic information, nursing knowledge, nursing attitude, and nursing practice on COVID-19 care. The questions were designed based on “COVID-19 diagnosis and treatment scheme (seventh edition),” “COVID-19 prevention and control program (sixth edition),” “COVID-19 laboratory testing technology guide (fifth edition)” and “COVID-19 psychological counseling work plan,” which were issued by the National Health Commission of China and were revised and verified by five frontline medical experts from a designated hospital of COVID-19.

The questionnaire is composed of two parts. The first part is general information including age, educational background, work experience, hospital rank, and workplace (outpatient clinics and emergency departments, fever clinics, isolation wards, and general wards). The second part assessed the KAP of nursing staff. The knowledge section includes suspected patient care, confirmed patient care, specimen collection, psychological care, and discharge guidance. There are 10 knowledge-related questions, 1 point for each correct answer, with a total score of 10 points. The attitude section includes a self-assessment of knowledge mastery, the desire to learn more knowledge, the influence of emotional state on nursing quality, and the ability and confidence to nursing care for COVID-19. Each question is scored on five levels, ranging from 1 point to 5 points. The total score is 20 points. The practice section includes implementing nursing in accordance with the nursing requirements of infectious diseases, preventing cross-infection consciously, changing daily manner of work to meet the needs during COVID-19, and adapting to psychological changes of patients actively. There are four nursing practice questions. Each question is scored on four levels, ranging from 1 point to 4 points. The total score is 16 points.

### Statistical Methods

Categorical data were described by numbers and percentages. The average scores of KAP were presented as median and interquartile range. Spearman rank correlation analysis or Kruskal–Wallis test was performed to analyze the relation between KAP scores and its factors. Ordered classification logistics regression analysis was performed to identify the risk factors affecting, and outcomes were presented as odds ratio and 95% confidence interval (CI). Data were analyzed using SPSS statistical software version 19.0. Two-tailed *P* value ≤ 0.05 was considered as statistically significant.

## Results

### Demographic Characteristics

In the study, a total of 7,716 nursing staff volunteered to participate in the survey; 7,716 valid questionnaires were returned. Of the participants, 47.8% were under 30 years old and 50.2% were aged from 31 to 49; 76.6% have an undergraduate education background; 58.9% have a working experience of <10 years; 60.7% are from municipal hospitals; 74.8% worked in general wards, 16.9% in outpatient clinics and emergency departments, 6.1% in isolation wards, and 2.2% in fever clinics (Table 1).

**Table 1:** The relation between demographic characteristics of responders and KAP scores.

**Characteristics**	**Demographic variables**	**Number**	**Percentage**	**Knowledge**		**Attitude**		**Practice**	
				**Statistics**	***P***	**Statistics**	***P***	**Statistics**	***P***
Age				0.143	<0.001	0.140	<0.001	0.096	<0.001
	≤ 30 years	3,689	47.81						
	31–49 years	3,870	50.16						
	≥50 years	157	2.03						
Educational level				0.114	<0.001	0.045	<0.001	0.013	0.240
	College degree or below	1,781	23.08						
	Undergraduate	5,908	76.57						
	Postgraduate	27	0.35						
Working experience				0.161	<0.001	0.145	<0.001	0.102	<0.001
	≤ 10 years	4,546	58.91						
	11–19 years	2,188	28.36						
	≥20 years	982	12.73						
Hospital rank				0.029	0.010	0.015	0.177	0.340	0.003
	County level	1,954	25.32						
	Municipal level	4,680	60.65						
	Provincial level	1,082	14.02						
Field of work				52.421^*^	<0.001	32.699^*^	<0.001	64.503^*^	<0.001
	Outpatient clinics and emergency departments	1,307	16.94						
	Fever clinics	169	2.19						
	Isolation wards	472	6.12						
	General wards	5,768	74.75						

* *is χ^2^, others are r_s_*.

### The KAP on COVID-19

The median and interquartile range of nursing knowledge, attitude, and practice on COVID-19 were 8 (2), 16 (3), and 14 (2), respectively.

The study shows that there is a high awareness rate among the respondents as to “patients' specimen collection,” “requirement of patients' temperature measuring before discharge,” “principles of placement in wards for suspected cases,” and “nursing procedure requirements for suspected cases,” with a correct answer rate of 99.0, 98.4, 95.8, and 95.8%, respectively. The correct rate of “frequency and duration of ventilation in wards,” “discharge standards for patients,” and “de-quarantine standards for suspected cases” was 73.4, 72.0, and 68.1%, respectively. The correct rate of “patients' psychological status” was only 14.3%.

Of the respondents, 65.3% believed that they had a good command of the COVID-19 nursing knowledge. The proportion of respondents with a strong desire to learn more nursing knowledge on COVID-19 was 74.3%. Facing the infectious disease, 17.5% of participants thought it had no impact on their nursing care quality; 66.4% thought they were neither panicked nor imprudent, and would try to ensure the nursing care quality; 9.3% indicated they had some anxiety and depression, which may affect their nursing quality and efficiency; 36.3% thought they were fully competent and confident to do a good job, while 47.6% thought they were competent and confident enough.

Of the respondents, 35.6% said they were fully able to care for COVID-19 patients according to the standards, 55.8% of them were basically able to handle in accordance with standards, and 8.5% said they were not yet good enough. In terms of consciously preventing cross-infection, 37.6% thought they did quite well and 53.8% thought they did good enough (Table 2).

**Table 2:** The score of every item in KAP.

**Variable**	**Item**	**scores**	**1**		**2**		**3**		**4**		**5**	
			***n***	**Constituent ratio (%)**	***n***	**Constituent ratio (%)**	***n***	**Constituent ratio (%)**	***n***	**Constituent ratio (%)**	***n***	**Constituent ratio (%)**
Knowledge		Total scores = 10										
	Specimen collection	0–1	7,639	99.00								
	Psychological care	0–1	1,100	14.26								
	Discharge guidance	0–2	1,474	19.10	6,218	80.59						
	Suspected patient care	0–3	267	3.46	2,564	33.23	4,880	63.25				
	Confirmed patient care	0–3	983	12.74	2,922	37.87	3,709	48.07				
Attitude		Total scores = 20										
	Knowledge mastery	1–5	0	0	25	0.32	2,653	34.38	4,586	59.43	452	5.86
	Learn more knowledge	1–5	5	0.06	4	0.05	207	2.68	1,929	25.00	5,571	72.20
	Emotional state	1–5	20	0.26	715	9.27	507	6.57	5121	66.37	1,353	17.53
	Confidence in nursing	1–5	6	0.08	71	0.92	1,163	15.07	3,674	47.62	2,802	36.31
Practice		Total scores = 16										
	Standardized care	1–4	7	0.09	657	8.51	4,302	55.75	2,750	35.64		
	Prevent cross infection	1–4	8	0.10	658	8.53	4,148	53.76	2,902	37.61		
	Change manner of work	1–4	1	0.01	69	0.89	1,891	24.51	5,755	74.59		
	Adapt to patient's psychological changes	1–4	6	0.08	211	2.73	2,078	26.93	5,421	70.26		

### Access to COVID-19 Nursing Knowledge

According to the survey, the main ways to acquire COVID-19 nursing knowledge were online video, training organized by hospitals, and website information. The proportion of respondents who acquired knowledge through the above three ways was 92.6, 88.6, and 74.9% respectively. Communication among colleagues, TV, and radio closely followed, with proportions of 64.9, 56.4, and 30.9%, respectively. The percentage of respondents who acquired knowledge through other means, such as newspapers, was lower (<20.0%).

#### Single-Factor Correlation Analysis of the Factors Affecting KAP Scores

We analyze the relationship between knowledge, attitude, and behavior. The results showed that knowledge was related to attitude (*r*_*s*_ = 0.083, *P* < 0.001) and behavior (*r*_*s*_ = 0.044, *P* < 0.001). Attitude was positively related to behavior (*r*_*s*_ = 0.553, *P* < 0.001).

In order to study the relationship between different factors and KAP scores, we conducted a single-factor rank correlation analysis. We found that age had a positive correlation with the scores of KAP, so did working experience. The correlation between workplace and the scores of KAP was also statistically significant. Educational background was not related to practice scores, but to knowledge and attitude scores. There was a positive correlation between hospital rank and knowledge score or practice score (Table 1).

#### Ordered Classification Logistics Regression Analysis to Identify the Influencing Factors of KAP Scores

Using quartile as cutoff point, the scores of KAP were divided into four grades and set as the dependent variable, respectively. All factors were included into independent variables.

Ordered classification logistics regression analysis showed that knowledge score of respondents aged under 30 or 11–49 is lower than that of respondents aged over 50 years; the OR value was 0.565 (95% CI: 0.397–0.803) and 0.625 (95% CI: 0.450–0.870). The result of work experience was consistent with age. The knowledge score of nursing staff in provincial hospitals was higher than both the scores in county level hospitals (OR = 0.729, 95% CI: 0.635–0.837) and in municipal level hospitals (OR = 0.754, 95% CI: 0.667–0.853). Nurses working in fever clinics and isolation wards scored higher than those in general wards, and the OR values were 1.657 (95% CI: 1.248–2.201) and 1.776 (95% CI: 1.491–2.116), respectively.

In terms of attitude, respondents aged ≥50 years scored higher than those aged ≤ 30 (OR = 0.630, 95% CI: 0.449–0.883). Respondents with working experience ≤ 10 years scored lower than those ≥20 years (OR = 0.654, 95% CI: 0.551–0.775). Respondents in isolation wards scored higher than those in general wards (OR = 1.542 95% CI: 1.298–1.832).

Respondents with work experience ≥20 years scored higher than those ≤ 10 years in practice scores (OR = 0.747, 95% CI: 0.629–0.886). Respondents working in provincial hospitals scored higher than those in county hospitals (OR = 0.815, 95% CI: 0.710–0.935). Workers from fever clinics and isolation wards have higher scores than those in general wards. The OR values were 1.367 (95% CI: 1.030–1.812) and 1.902 (95% CI: 1.590–2.274), respectively (Table 3).

**Table 3:** Ordered classification logistics regression analysis of the risk factors about KAP scores.

**Dependent variable**	**Characteristics**		**Reference**	**β**	**S.E**.	**Wald Chisq**	***P***	**OR**	**95% CI**
Knowledge	Age	≤ 30	≥50	−0.572	0.180	10.137	0.001	0.565	0.397–0.803
		31–49	≥50	−0.469	0.169	7.766	0.005	0.625	0.450–0.870
	Working time	≤ 10	≥20	−0.713	0.088	64.985	<0.001	0.490	0.412–0.583
		11–19	≥20	−0.488	0.076	40.751	<0.001	0.614	0.529–0.713
	Hospital rank	County	Province	−0.316	0.071	20.095	<0.001	0.729	0.635–0.837
		City	Province	−0.282	0.063	20.078	<0.001	0.754	0.667–0.853
	Field of work	Fever clinic	General ward	0.505	0.145	12.175	<0.001	1.657	1.248–2.201
		Isolation ward	General ward	0.575	0.089	41.447	<0.001	1.776	1.491–2.116
Attitude	Age	≤ 30	≥50	−0.462	0.172	7.188	0.007	0.630	0.449–0.883
	Working time	≤ 10	≥20	−0.425	0.087	23.968	<0.001	0.654	0.551–0.775
	Field of work	Isolation ward	General ward	0.433	0.088	24.329	<0.001	1.542	1.298–1.832
Practice	Working time	≤ 10	≥20	−0.292	0.087	11.188	0.001	0.747	0.629–0.886
	Hospital rank	County	Province	−0.205	0.070	8.575	0.003	0.815	0.710–0.935
	Field of work	Fever clinic	General ward	0.312	0.144	4.701	0.030	1.367	1.030–1.812
		Isolation ward	General ward	0.643	0.091	49.584	<0.001	1.902	1.590–2.274

## Discussion

The number of people diagnosed with COVID-19 was increasing continuously. The appearance of COVID-19 was listed by WHO as a “Public Health Emergency of International Concern” (PHEIC) (5), which is the highest level of infectious disease emergency response within the WHO system. It is important to staff enough nurses to care for the infected and ensure the quality of care in major public health emergencies (6). Our research was conducted during the new coronary pneumonia epidemic and the sample size was large, which is representative of the KAP of nursing staff regarding COVID-19.

The results of our study showed that nurses working in isolation wards have higher KAP scores than those in general wards. The KAP scores in outpatient and emergency departments were not so different from those in general wards. For sudden infectious diseases, the workload of nursing staff is large, and they face the risk of infection and bear greater psychological pressure than doctors (7). A study showed that non-frontline workers had lower confidence in defeating the virus compared to frontline HCWs (8). Acquiring knowledge, generating beliefs, and forming behavior are three consecutive processes (9). Higher scores of KAP help avoid occupational exposure and prevent nosocomial infections among nursing staff. Of the infected health care workers who were presumed to have been infected in hospital, 31 (77.5%) were from general wards, 7 (17.5%) were from the emergency department, and 2 (5%) were from the ICU (10). This may be related to a lack of knowledge or awareness of protection among nursing staff in general wards.

Another important influencing factor we found was working experience, which was significantly associated with the KAP scores. Nurses who worked more than 20 years have a significantly higher score than those who worked <10 years. Our result is similar to other studies (11, 12). The richer his/her work experience, the more confident is the nurse to face and deal with public health emergencies.

We found that the knowledge and behavior scores of respondents from provincial units were higher than those from county units. The difference may be partly because of necessary equipment and protective measures against infection in provincial-level health institutions, as well as the higher accessibility of relevant information and training on COVID-19.

The theory model of KAP is widely used in nursing work. Knowledge is the basis of behavior change, which is also the decisive factor. This study found that the scoring rate of psychological nursing was only 14.3%. In terms of knowledge on COVID-19, 59.4% of the nursing staff showed that they had a better grasp of relevant knowledge, only 5.9% showed that they had fully mastered it, and 72.2% of the nursing staff said they hoped to continue to learn more about the new coronary pneumonia. Knowledge is a prerequisite for establishing prevention beliefs. Compared with health problems, nursing staff are more likely to ignore the psychological needs of patients. When facing an unknown disease without any specific medicine to treat, patients especially the elderly may have negative emotions such as anxiety and depression. Nurses who have the necessary psychological knowledge and consciously perform psychological nursing work can help patients recover better.

For goodness of fit of the predictor model on practice, the *R*^2^ = 0.021 (Cox and Snell) is rather low to describe determinant factors; other factors need to be explored in future research.

## Data Availability Statement

The raw data supporting the conclusions of this article will be made available by the authors, without undue reservation.

## Ethics Statement

The studies involving human participants were reviewed and approved by Medical Ethic Committee, School of Public Health, Zhejiang University.

## Author Contributions

XW, FW, XL, and HG: each author has been sufficiently involved in this submission to take public responsibility for the work, meaning that each author has made substantial contributions to the conception and design of the study. HG and XW: acquisition of data. FW and XL: analysis and interpretation of the data. XW, FW, XL, and HG: drafting the article and revising it critically for important intellectual content. All authors contributed to the article and approved the submitted version.

## Conflict of Interest

The authors declare that the research was conducted in the absence of any commercial or financial relationships that could be construed as a potential conflict of interest.
